# Exercise repetition rate measured with simple sensors at home can be used to estimate Upper Extremity Fugl-Meyer score after stroke

**DOI:** 10.3389/fresc.2023.1181766

**Published:** 2023-06-19

**Authors:** Veronica A. Swanson, Christopher A. Johnson, Daniel K. Zondervan, Susan J. Shaw, David J. Reinkensmeyer

**Affiliations:** ^1^ Biorobotics Laboratory, Department of Mechanical and Aerospace Engineering, University of California, Irvine, Irvine, CA, United States; ^2^ Biorobotics Laboratory, Department of Biomedical Engineering, University of California, Irvine, Irvine, CA, United States; ^3^ Flint Rehab, LLC, Irvine, CA, United States; ^4^ Department of Neurology, Rancho Los Amigos National Rehabilitation Center, Downey, CA, United States; ^5^ Department of Anatomy and Neurobiology, UC Irvine School of Medicine, University of California, Irvine, Irvine, CA, United States

**Keywords:** assessment, stroke, sensors, mRehab, Fugl-Meyer, rehabilitation, home, remote

## Abstract

**Introduction:**

It would be valuable if home-based rehabilitation training technologies could automatically assess arm impairment after stroke. Here, we tested whether a simple measure—the repetition rate (or “rep rate”) when performing specific exercises as measured with simple sensors—can be used to estimate Upper Extremity Fugl-Meyer (UEFM) score.

**Methods:**

41 individuals with arm impairment after stroke performed 12 sensor-guided exercises under therapist supervision using a commercial sensor system comprised of two pucks that use force and motion sensing to measure the start and end of each exercise repetition. 14 of these participants then used the system at home for three weeks.

**Results:**

Using linear regression, UEFM score was well estimated using the rep rate of one forward-reaching exercise from the set of 12 exercises (r^2^ = 0.75); this exercise required participants to alternately tap pucks spaced about 20 cm apart (one proximal, one distal) on a table in front of them. UEFM score was even better predicted using an exponential model and forward-reaching rep rate (Leave One Out Cross Validation (LOOCV) r^2^ = 0.83). We also tested the ability of a nonlinear, multivariate model (a regression tree) to predict UEFM, but such a model did not improve prediction (LOOCV r^2^ = 0.72). However, the optimal decision tree also used the forward-reaching task along with a pinch grip task to subdivide more and less impaired patients in a way consistent with clinical intuition. At home, rep rate for the forward-reaching exercise well predicted UEFM score using an exponential model (LOOCV r^2^ = 0.69), but only after we re-estimated coefficients using the home data.

**Discussion:**

These results show how a simple measure—exercise rep rate measured with simple sensors—can be used to infer an arm impairment score and suggest that prediction models should be tuned separately for the clinic and home environments.

## 1. Introduction

Recovery from stroke is a long process requiring extended periods of neurologic rehabilitation, which includes cycles of assessment, prescribed interventions, evaluation, and adjustment of interventions (1, 2). The assessment stage is crucial to determine a patient's treatment plan and to evaluate the effectiveness of that plan after its execution. There are a variety of standardized assessments for stroke rehabilitation in practice and research (3, 4) that span the domains of impairments, functional limitations, and barriers to participation a stroke survivor might face.

Any assessment used must have appropriate psychometric properties (4–6), including validity, reliability, and responsiveness. A particularly important goal is that the assessment works well when evaluators change, i.e., assessments should have good inter-rater reliability (7). Common motor impairment assessments with good psychometric properties used in stroke rehabilitation for the upper extremity include the Upper Extremity Fugl-Meyer (UEFM) Test, the Box and Block (BB) test, the Action Research Arm Test, the Nine Hole Peg Test, and the Wolf Motor Function Test (5).

A growing goal in rehabilitation research and development is to automate clinical assessments to reduce the burden on clinicians' time (8), eliminate the potential influence of evaluator subjectivity, improve the quality and access to effective remote care (9, 10) and support self-directed continuation of rehabilitation at home (1).

Home rehabilitation has multiple goals including reducing inpatient stays through early supported discharge, continuing rehabilitation at home to replace institutional rehabilitation, and providing home exercise programs to help patients maintain or augment the gains made under supervision of a health care professional (11). Studies on home rehabilitation have shown comparable outcomes for patients pursuing rehabilitation at home and patients in institutional care. In some cases, early supported discharge promoted community reintegration and reduced costs of care more than institutionalized patients (12). For people living at home continuing their care, a systematic review found significant effects in favor of home-based rehabilitation on functional independence measures, and some studies found cost benefits and increased caregiver satisfaction for individuals receiving home-based rehabilitation (13).

Exercise is important for improving functional capacity, performance of activities of daily living, and quality of life for post-stroke individuals (14) and may reduce the risk of stroke recurrence (15). Achieving the American Heart Association's recommendations of performing aerobic exercises 3 to 7 days per week and strengthening, flexibility, and neuromuscular exercise 2 to 3 days a week is difficult to achieve in institutionalized care and could be better realized through home exercise programs. However, maintaining motivation to adhere to home exercise programs is difficult for many stroke survivors (11), with rates of apathy in stroke survivors above 30% (16) and evidence that apathy has a strong effect on limiting participation in meaningful activity (17). Successfully administering assessments in the home environment could support patient motivation by tracking recovery progression and could be used by healthcare providers to adjust aspects of treatment plans, such as the specific tasks being used, without or in between in-person encounters.

The assessments previously mentioned are performance measures, in which a patient performs specific motions or activities, and the assessment is designed to analyze the body function or evaluate the execution of the activity (3, 7). These assessments are therefore prime candidates for automation, as a patient could perform the assessment activity independently, a sensorized system could record data during performance of the activity, and an algorithm could generate a score for the activity, approximating as closely as possible, the score that a trained clinical evaluator would give the patient as part of the assessment. This is preferable to creating new assessments designed specifically for technical automation due to the aforementioned psychometric requirements, which involves an extensive process of design and clinical validation (7). This work focuses on the Upper Extremity Fugl-Meyer (UEFM) (18) because it is a widely used measure in stroke rehabilitation research showing both high reliability and validity (19). The assessment was developed to consider classically defined patterns of stroke recovery where motor function first returns in proximal muscles before distal muscles, and flexor synergistic movements return before extensor synergies in the arms (19–21).

Previous efforts to objectively measure and automate the UEFM (see Table 1) have used Image Processing Systems such as the Microsoft Kinect (28), Inertial Measurement Units (IMUs) (24), and mechanical systems such as flex sensors, or a combination of these technologies (29). However, image-based systems can suffer from variability due to environmental lighting and visual clutter. IMUs and mechanical systems are frequently used in wearable configurations, often requiring precise placement of multiple sensing units, which is difficult to do independently for patients with impairments. Two strategies commonly employed are to instrument the assessment, whereby patients perform the assessment or a subset of the assessment's items and data is recorded during the performance, or estimate an assessment score from data taken during representative motions or functional tasks (7). Calculating a total, continuous value rather than estimating individual line items of an assessment could provide an advantage over conventional calculation methods which frequently rely on ordinal measures, which are less sensitive to smaller changes and potentially less precise than a continuous-valued output (30). Using these methods, prediction strength has ranged from r^2^ of 0.21 to 0.97 (see Table 1).

**Table 1:** Methods to predict fugl-Meyer assessment scores using sensors. Abbreviations following (7), IMU, inertial measurement units; EEG, electroencephalogram; MMS, mechanical systems; IMS, image processing systems; OMS, optoelectronic systems. N in the table is the number of participants in each study.

Reference	Sensors	N	Task	Evaluation Features	Prediction/Estimation Strength
(22)	4 IMUs and EMG	34	Voluntary Upward Reaching	Max Shoulder Joint Angle, Peak and Average Arm Speed, Torso Balance Muscle Synergy	All Features were significant (*p* < 0.001, r^2^ > 0.34
(23)	4 IMUs	37	Finger-To-Nose	Movement time (MT), mean velocity (VM), peak velocity (VP), percentage of time to peak velocity (TVP%), number of movement units (NMU), and normalized integrated jerk (NIJ)	VP,VM,NMU (*p* < 0.05, r^2^ > 0.42)
(8)	IMS	10	26 UEFM items	Joint Angle, Segment Rotation, Landmark Position	r^2^ = 0.985
(24)	6 IMUs	24	Selected tasks from WFMT	20 speed, smoothness and coordination features	r^2^ > 0.44
(25)	OMS	34	Reaching Task	ROM, Movement Smoothness, Trunk displacement, Trunk forward inclination	All Features were significant except Shoulder ROM (*p* < 0.001, r^2^ > 0.21)
(26)	9 IMU	26	Isolated shoulder flexion, pointing, reach-to-grasp a glass, and key insertion	joint ranges of shoulder abduction/adduction, shoulder flexion/extension, and elbow flexion/extension; trunk displacement; shoulder–elbow correlation coefficient; median slope; and curve efficiency	r^2^ > 0.24
(27)	MMS	82	4 shoulder-elbow tasks, 3 wrist and forearm tasks	24 kinematic metrics for the shoulder-elbow, 35 metrics for wrist and forearm	r^2^ = 0.67 (*p* < 0.001) for the linear model and r^2^ = 0.77 (*p* < 0.001) for the nonlinear model

Using the rate of task performance to estimate impairment is not unique. For example, it is the strategy used by the Box and Blocks assessment. However, it is a strategy not often explored in attempts to sensorize and automate clinical assessments. This strategy potentially allows for a simple sensor array to be used, since it need only count reps, and a small number of test items [i.e., the task(s) whose rep rate is assessed]. These are desirable features for an assessment procedure intended for patients to autonomously execute in the home.

The goal of this work was to determine how well we could estimate the UEFM score for individuals who have experienced a stroke based on their exercise rep rate as they interacted with a sensorized home-rehabilitation system. We used the rep rates of tasks completed with the system, rather than raw sensor data, which is a method that could be easily implemented with other systems. The data we used included data from a recently published randomized controlled trial (RCT) of the sensor system (31).

## 2. Methods

### 2.1. FitMi overview

**Figure 1:**
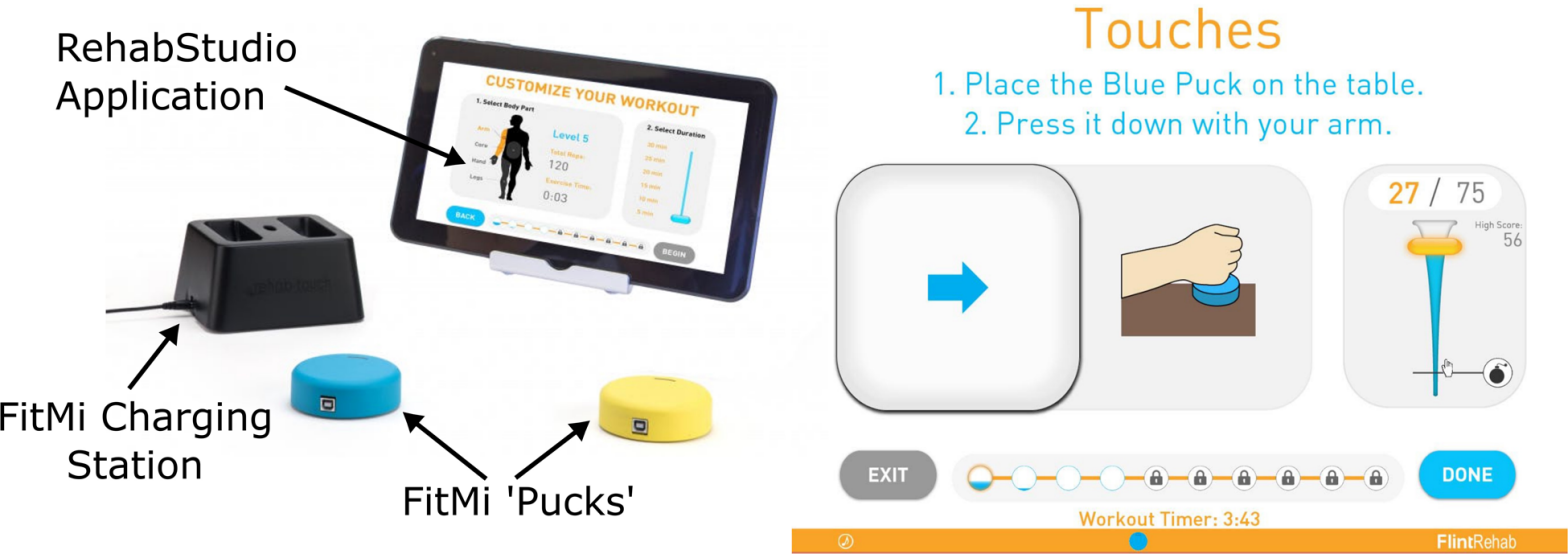
FitMi (produced by flint rehab, LLC) consists of two force and motion sensing pucks and a companion software application. Left: FitMi hardware. Right: an example of the FitMi software interface during an exercise. Note, FitMi can be used with a custom 10” touchscreen tablet in a kiosk mode (shown) or with an individual's existing computing hardware using a Bluetooth receiver.

The FitMi system (Flint Rehab, LLC) consists of two wireless pucks that each contain an accelerometer, gyroscope, magnetometer, load cell, light emitting diode (LED), and a vibration motor (Figure 1). Custom software, run on a personal computer or tablet, presents a set of exercises for users to complete (Supplementary Text S1). A total of 40 exercises are available in the system, with 10 each designed for the legs, core, arms, and hands. During the exercises, a universal serial bus (USB) receiver collects sensor data from the pucks and the software shows how to move the pucks or move between each puck to start and finish a repetition of the activity, reacting to the changing position or state of the pucks indicated by the sensors. For each exercise, users are presented a target number of repetitions and a limited amount of time to complete them. A small amount of time is added for each repetition completed, encouraging users to perform repetitions at a desired rate. As users complete the target number of repetitions presented, the challenge of the experience is increased by increasing the target number of repetitions and making more difficult exercises available.

### 2.2. Experimental protocol

Data for this study came from two experiments. The first experiment, the “In-Clinic Experiment”, had two goals: (1) Collect data from a broad population of stroke patients to evaluate usability of the system and to facilitate exploratory analysis; and (2) Screen participants for the second experiment. The second experiment was a randomized controlled trial (RCT) comparing home-based therapy with FitMi to conventional therapy for individuals in the subacute phase of stroke was performed at Rancho Los Amigos National Rehabilitation Center in Downey, CA from November of 2018 to March of 2020 (ClinicalTrials.gov #NCT03503617) (31). The in-clinic experiment included participants in the chronic phase and sub-acute phase of stroke recovery, while the subsequent RCT contained only sub-acute participants who met the following inclusion criteria: aged 18 to 85 who experienced one or more strokes between 2 weeks and 4 months prior with a baseline Upper Extremity Fugl-Meyer (UEFM) Score >5 and ≤55 out of 66. For individuals who continued on to participate in the RCT following the in-clinic experiment, the in-clinic experiment served as their baseline assessment.

Individuals who participated in the in-clinic experiment were first guided through a set of 12 exercises (A4: Reach to Target #2, A6: Wrist Supination, A7: Bicep Curls, C4: Twists, C7: Oblique Crunch, C8: Standard Crunch, H3: Gripping, H5: Key Pinch Grip, H10: Object Flipping, L1: Stomps, L5 Marching, L9: Ankle Rotation; See Supplementary Text S1) in the FitMi system by a rehabilitation therapist. A single therapist conducted the in-clinic assessment for each participant. For each exercise, the therapist ensured a standard placement of the pucks across all participants according to the instructions presented in the system. The therapist also instructed participants how to perform the exercise correctly. They then verified that the participant could perform the exercise without undesired compensation patterns (i.e., any movement patterns that could risk injury or maladaptive plasticity if performed several times in succession). If a participant was unable to complete an exercise or unable to perform the exercise without compensation, the therapist recorded that the participant performed zero repetitions of that exercise and moved on to the next exercise. Otherwise, the therapist instructed them to complete as many repetitions of the exercise as they could in 45 s and recorded the number of repetitions performed. Participants were given up to 2 min to rest between exercises.

Of the 41 participants of the in-clinic experiment, 27 participants, who met the inclusion criteria and agreed to participate, received therapy as part of the RCT. In the RCT, they were randomized into a FitMi group or a Conventional Therapy group using adaptive randomization to ensure matched levels of impairment between the groups. To accomplish this, subjects were classified by their UEFM Score into 3 levels (i.e., 5–22, 23–39, 40–55) and then randomized by alternating block allocation (32). Participants in both groups were instructed to perform self-guided therapy at home for at least three hours/week for three consecutive weeks. The FitMi group performed their therapy using the FitMi system, and the Conventional Therapy group used a paper booklet of exercises. During the at-home phase of the study, exercise instructions and recommended puck placements were provided for each exercise in written instructions and in a video that participants could view before the exercise. However, beyond these instructions, standardization of the puck placement was left to the participant. Participants’ activity in the FitMi system was recorded, including the date and time an exercise was performed, the type of exercise, the number of repetitions completed, the amount of time spent performing the exercise, and the difficulty level at which the exercise was performed. At the start of the trial, the three easiest exercises from each body region were available at the lowest difficulty level. After three weeks, each participant returned for an end-of-therapy assessment, and then again after one month for a follow-up assessment. The Conventional Therapy Group's data (*n* = 13) from the end-of-therapy and follow-up assessments are not used in the present study as they did not use the FitMi system during the home-therapy they performed during the RCT.

#### 2.2.1. Clinical assessments

Therapists performed a battery of clinical assessments during the in-clinic experiment including the Upper Extremity Fugl-Meyer (UEFM) (18), Box and Blocks Test (33), the 10 Meter Walk Test (34), the Modified Ashworth Spasticity (MAS) scale (35) for the elbow, wrist, and fingers, the Visual Analog Pain (VAP) scale (36) for the upper extremity, Trunk Impairment Scale (37), Shoulder Subluxation using thumb widths (38), and Mini Mental Status (39). Several measures were taken during the end-of-therapy and follow-up assessments of the subsequent RCT. From the end-of-therapy and follow-up assessments, only the UEFM scores of the FitMi group taken during their end-of-therapy assessment are used in this study.

### 2.3. Statistical analysis

In the introductory session, all participants performed each exercise for 45 s, if they were able to perform the exercise. We converted the number of repetitions completed to the rate at which repetitions were completed. For analysis, the MAS was split between the different categories measured (elbow extension, elbow flexion, wrist extension, wrist flexion, finger extension, and finger flexion), and items scored with a “+” were transformed to a numerical quantity by adding 0.5 to facilitate analysis. Unless otherwise mentioned, all analyses were performed in Matlab 2020b (40).

#### 2.3.1. Clinic data analysis

As an exploratory analysis, linear regression was used to model the relationship between each exercise performed and each assessment taken during the in-clinic experiment (*n* = 41). A Belsley test was used to confirm there was no collinearity between the exercises and assessments. Several nonlinear functions were fit to the rate and outcome data for the pair which presented the strongest relationship from the previous step. The goodness of the fit for each candidate function was evaluated by comparing the resulting root mean squared error (RMSE), r-squared, and appropriateness of the function for the data as determined by the study team. Finally, the best fitting model was validated using a leave one out cross validation (LOOCV) procedure.

#### 2.3.2. Home data analysis

The subset of participants who were randomized to the FitMi treatment group of the RCT (*n* = 14) took the system home for 3 weeks to use without supervision. To test the suitability of the selected model for estimating clinical scores using exercise data from participants' home-activity, exercise data from these participants were used to estimate their clinical scores using the strongest model identified by the curve fitting process described above. The model was then refit to the home exercise data and participants’ UEFM scores from the end-of-therapy assessment of the RCT to improve performance and validated following LOOCV. Due to one participant not performing the “A4: Reach to Target 2” exercise at home, analyses for home data were performed without this participant (*n* = 13).

#### 2.3.3. Decision tree

To incorporate multiple exercises into a single explanatory, nonlinear model, regression trees were fit to the data taken during the in-clinic experiment (*n* = 41) using the Decision Tree Regressor from Scikit Learn in Python. As the data set is smaller than typical for machine learning applications, and the goal of this model was explanatory rather than predictive, the entire data set was used for training models, and models were evaluated using metrics from the training data. Decision tree models can be prone to overfitting, where the generated model might describe noise of the training set more than any underlying generalizable phenomena present. To prevent overfitting, multiple models were fit with varying maximum allowable depths and minimum samples per leaf (i.e., prediction node), where increasing the depth and reducing the minimum samples per leaf results in models with increasing accuracy but also increasing potential for overfitting. Models were compared by the research team considering the complexity of the resulting model, the depth and leaf design criteria, the RMSE, and the r^2^. The results of this process are shown in Supplementary Figure S1. The final selected model was validated following LOOCV.

## 3. Results

### 3.1. Participants

**Figure 2:**
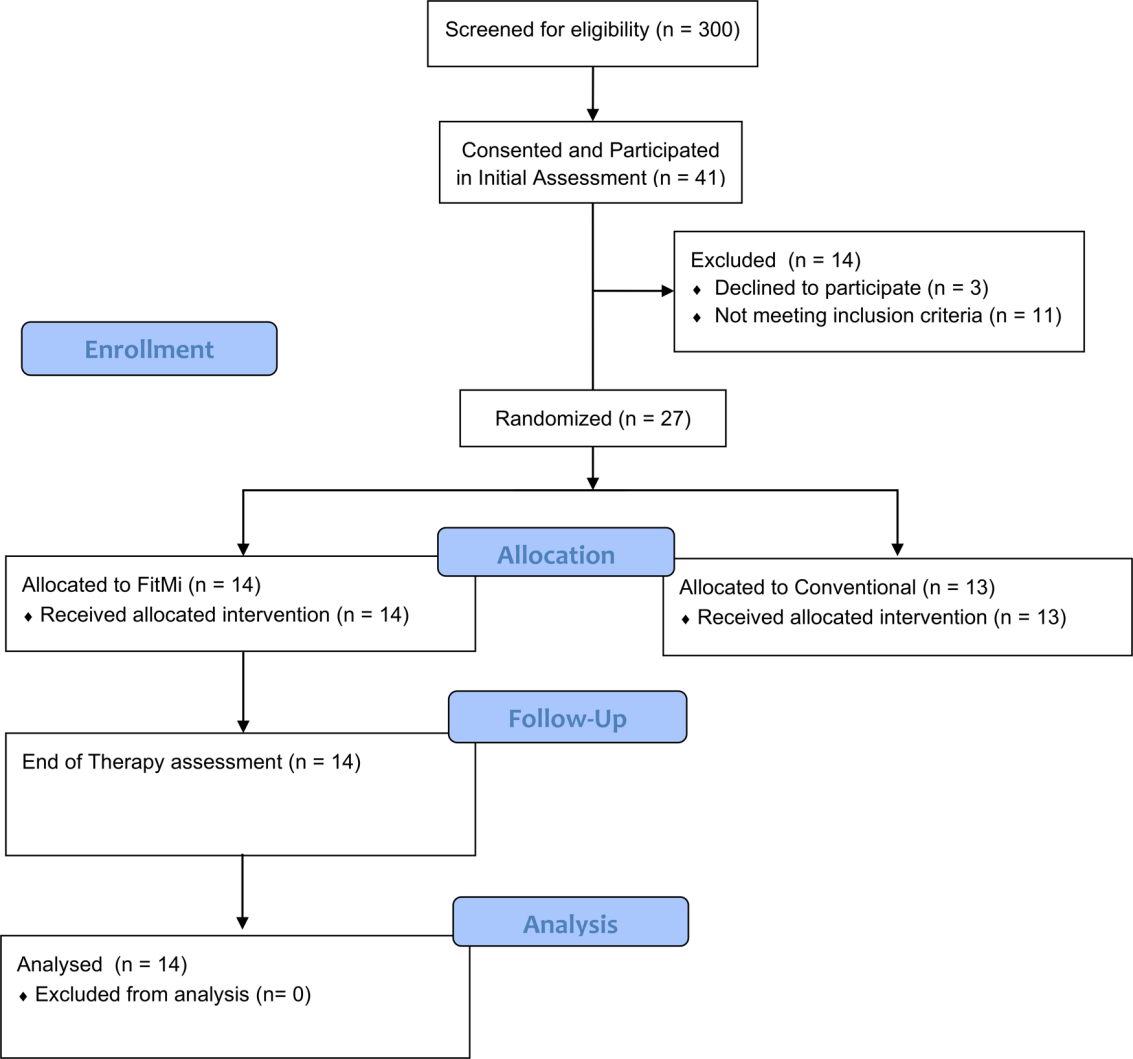
Participant flow diagram detailing screening, allocation, and assessments for individuals in the in-clinic experiment and the RCT. Follow-up data for individuals allocated to the conventional therapy group of the RCT was not used in this study.

Participants were recruited from November 20, 2018 to March 12, 2020. 41 individuals participated in the in-clinic experiment, and 27 of the participants from the in-clinic experiment moved on to the clinical trial, during which 14 participants were randomized to the FitMi group (Figure 2). Participants' clinical characteristics are shown in Table 2. All participants were right hand dominant.

**Table 2:** Demographics of participants. All participants are included in the “Clinic Data” column, and the subset of participants who continued to the FitMi group are included in the “Randomized Control Trial” column. For each assessment, we list the minimum and maximum score possible in the left column, if the assessment has such limits. In the right columns, values are reported as Mean ± SD, [minimum, maximum] or Score (Number of Participants) as appropriate.

	In-Clinic Experiment	Randomized Controlled Trial
Number of Participants	41	14
Age (years)	52.3 ± 10.1	50.3 ± 10.9
Sex (M/F)	33/8	14/0
Stroke Type (I, H, B)	30 I, 10 H, 1 B	11 I, 3 H
Impaired Side (L/R)	27 L, 14 R	10 L, 4 R
Weeks Post Stroke	66 ± 114, [4.3, 456.7]	9.7 ± 4.5, [4.3, 17.9]
Mini Mental Status [min and max possible: 0 30]	29.56 ± 0.71, [27, 30]	29.36 ± 0.63, [28, 30]
Upper Extremity Fugl-Meyer [0 66]	33.4 ± 15.4, [9, 58]	36.7 ± 15.4, [12, 53]
Box and Blocks* [0 150]	19.1 ± 17.2, [0, 50]	25.4 ± 17.6, [0, 50]
Trunk Impairment [0 27]	17.61 ± 2.68, [13, 23]	18.36 ± 2.27, [15, 23]
10 Meter Walk (m/s)	0.87 ± 0.32, [0.21, 1.50]	0.98 ± 0.37, [0.23, 1.50]
Visual Analog Pain [0 10]	0 (24), 1 (3), 2 (4), 3 (4), 4 (5), 5 (1)	0 (8), 1 (1), 2 (1), 3 (1), 4 (3)
**Modified Ashworth Scale –Extension [0 4]**		
Elbow	0 (25), 1 (8), 1.5 (5), 2 (3)	0 (9), 1 (2), 1.5 (2), 2 (1)
Wrist	0 (37), 1 (4)	0 (13), 1 (1)
Fingers	0 (40), 1 (1)	0 (14)
**Modified Ashworth Scale –Flexion [0 4]**		
Elbow	0 (14), 1 (14), 1.5 (5), 2 (6), 3 (2)	0 (3), 1 (8), 1.5 (1), 2 (2)
Wrist	0 (13), 1 (11), 1.5 (8), 2 (8), 3 (1)	0 (5), 1 (2), 1.5 (5), 2 (2)
Fingers	0 (17), 1 (10), 1.5 (6), 2 (6), 3 (2)	0 (5), 1 (4), 1.5 (3), 2 (2)
Shoulder Subluxation (expressed as thumb widths)	0 (34), 0.5 (1), 1 (5)	0 (12), 1 (2)

M, male; F, female; I, Ischemic; H, hemorrhagic; B, both Ischemic and hemorrhagic; L, left; R, right.

*The average Box and Blocks Test score for unimpaired participants who are age-matched to the mean age of our participants is 69 (41).

### 3.2. Heatmap

**Figure 3:**
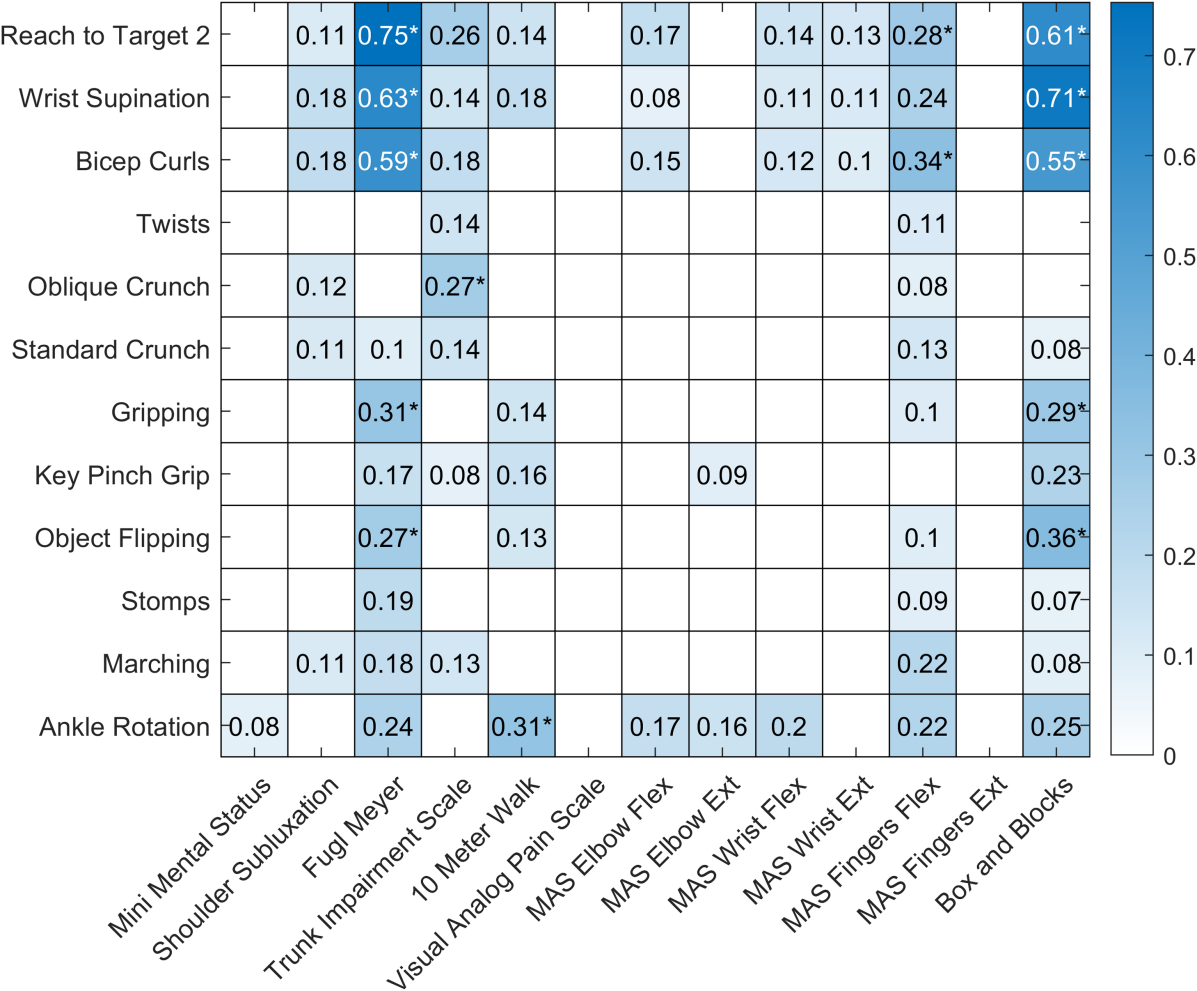
Heatmap showing the results of the regression analyses for each exercise and outcome pair. Regressions with an F-statistic *p* value > 0.05 are shown with a white box. r^2^ values for regressions with an F-statistic *p* value ≤ 0.05 are shown in blue, with darker colors indicating stronger relationships. Regressions with a *p* value below the Bonferroni adjusted alpha value (0.00032) are indicated with an asterisk.

Figure 3 shows the results of the regression analyses exploring the relationship between each exercise performed and each outcome measured. Only regressions with an F-statistic *p* value ≤ 0.05 are shown, and regressions with a *p* value below the Bonferroni adjusted alpha value (0.00032) are indicated with an asterisk. The UEFM and Box and Blocks Test scores were strongly correlated with the rep rates from three of the exercises.

### 3.3. Curve fitting

**Figure 4:**
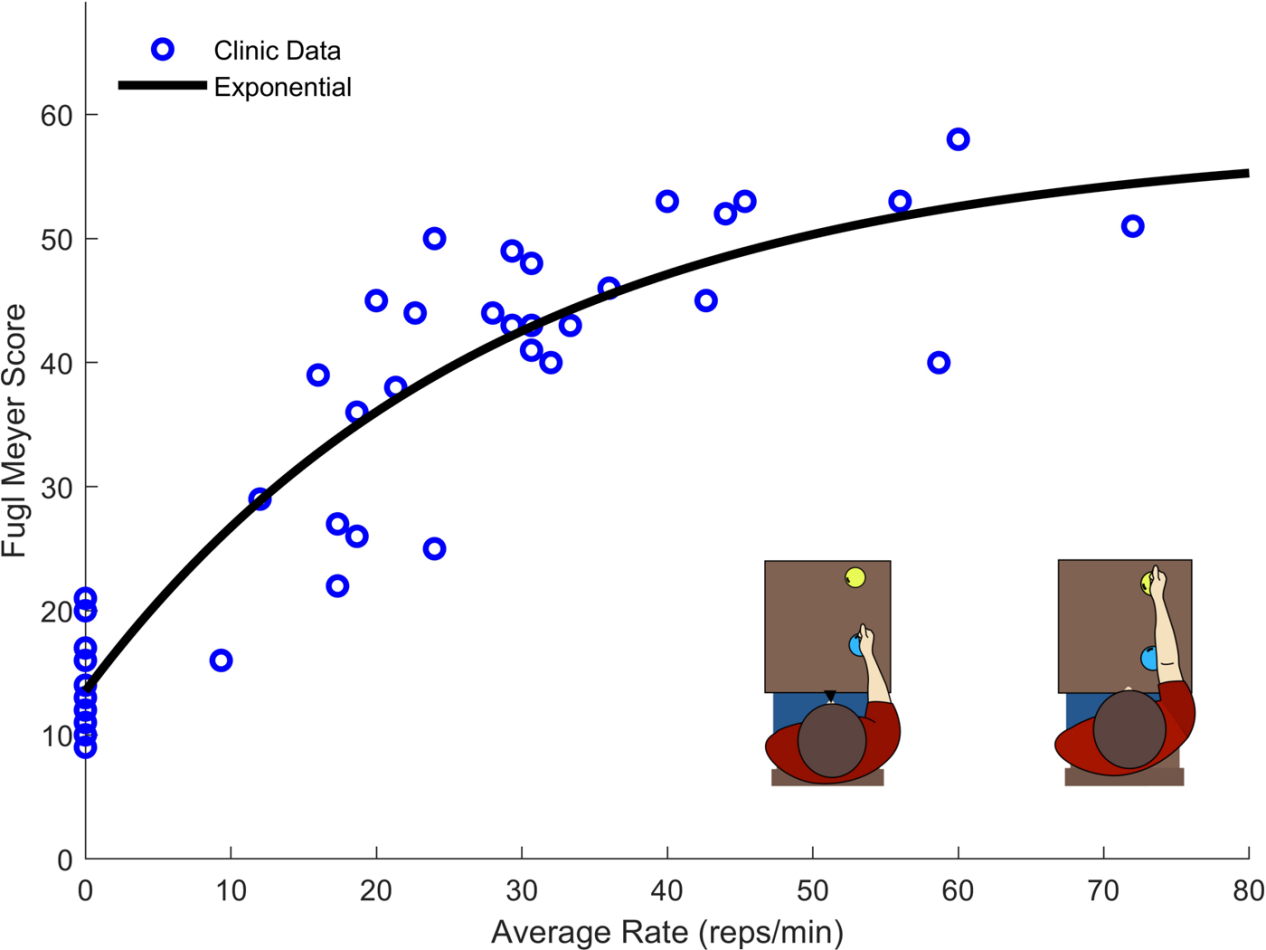
Exercise rate and UEFM data taken from 41 participants performing the “A4: Reach to Target #2”. The exponential function provided the best fit while providing an asymptotic structure which well describes the maximum score of the UEFM.

The strongest correlation was present for the regression analysis between “A4: Reach to Target 2” and the Upper Extremity Fugl-Meyer (UEFM) assessment (adjusted r^2^ = 0.75, *p* value < 0.001). To better model this relationship, we fit a second order polynomial, a power function, a logarithmic function, and an exponential function to the data (Figure 4).

Though the polynomial function produced better fit statistics (r^2^ = 0.86, RMSE = 5.92), the exponential fit (r^2^ = 0.85, RMSE = 6.00) was selected for its asymptotic structure, which is consistent with the fact that the UEFM score has a maximum. The final LOOCV resulted in an exponential model with r^2^ = 0.83, RMSE = 6.22.

### 3.4. Home data

**Figure 5:**
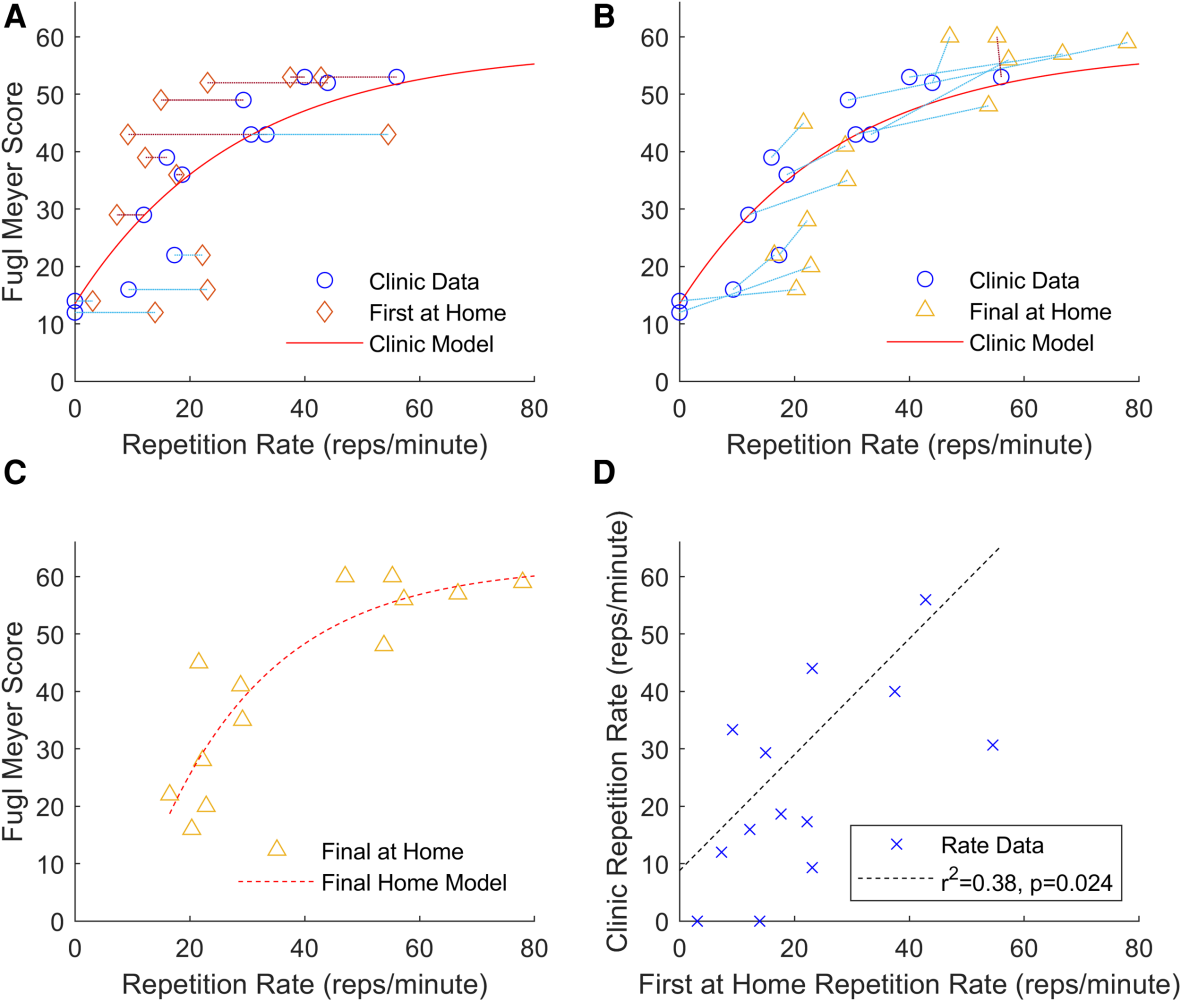
(**A**) Data from participants’ first performance at home of the “A4: Reach to Target #2” exercise plotted against their clinic exercise data and the model generated from the clinic data. (**B**) Data from participants’ last performance at home of the “A4: Reach to Target #2” exercise and their UEFM score taken at the end-of-therapy assessment plotted against their clinic exercise data and the model generated from the clinic data. In both figures (**A**) and (**B**), triangles are used to indicate exercises performed at home, circles are used to indicate exercises performed in the clinic, and dashed lines are used to connect each participant's clinic data and their respective home data. Red lines indicate that the participant's exercise rate slowed compared to their clinic performance, and blue lines indicate that the participant's exercise rate increased compared to their clinic performance. (**C**) A model with the same functional form as the model developed with the clinic data was fit using only the data from participants’ last performance at home of the “A4: Reach to Target #2” exercise and their UEFM scores taken at the end-of-therapy assessment. (**D**) Comparison of participants’ first performance at home of the “A4: Reach to Target #2” exercise plotted against their clinic exercise data evaluated using a linear regression.

To validate the model for use in the home setting, the exponential model shown in Figure 4 was used to estimate participant's UEFM scores using the exercise rate data from participants' first performance of the “A4: Reach to Target #2” exercise at home. Though participants' rates in their first home performance were correlated with their rates performed in the clinic (r = 0.62), the resulting fit was lower quality than for the data collected in the clinic (Clinic Model with Clinic Data: r^2^ = 0.85, RMSE = 6.00; Clinic Model with First Home Data: r^2^ = 0.24, RMSE = 12.81). In their first at-home performance, more severely impaired participants tended to speed up and less severely impaired participants tended to slow down relative to their in-clinic performance (Figures 5A,D). This change in exercise rate was correlated with participants' initial UEFM scores (r2 = 0.38). While data from participants' last performance of the “A4: Reach to Target #2” at home exercise did not fit the clinic-based model well, they appeared to follow a more consistent pattern than the data from participants' first performance (Figure 5B). The final home performance and end-of-therapy UEFM data resulted in improved fit statistics compared to the first home performance paired with in-clinic UEFM scores, but results were still lower quality than for the data generated in the clinic (Clinic Model with Clinic Data: r^2^ = 0.85, RMSE = 6.00; Clinic Model with Final Home Data: r^2^ = 0.50, RMSE = 11.17). Fitting a model of the same structure to the final performance of “A4: Reach to Target #2” at home and the UEFM scores taken at end-of-therapy resulted in a model with scores closer to the clinic-generated model (Final Home Model with Final Home Data: r^2^ = 0.80, RMSE = 8.09). Performing LOOCV on this final model produced a model with r^2 ^= 0.69, RSME = 8.70.

### 3.5. Decision tree

**Figure 6:**
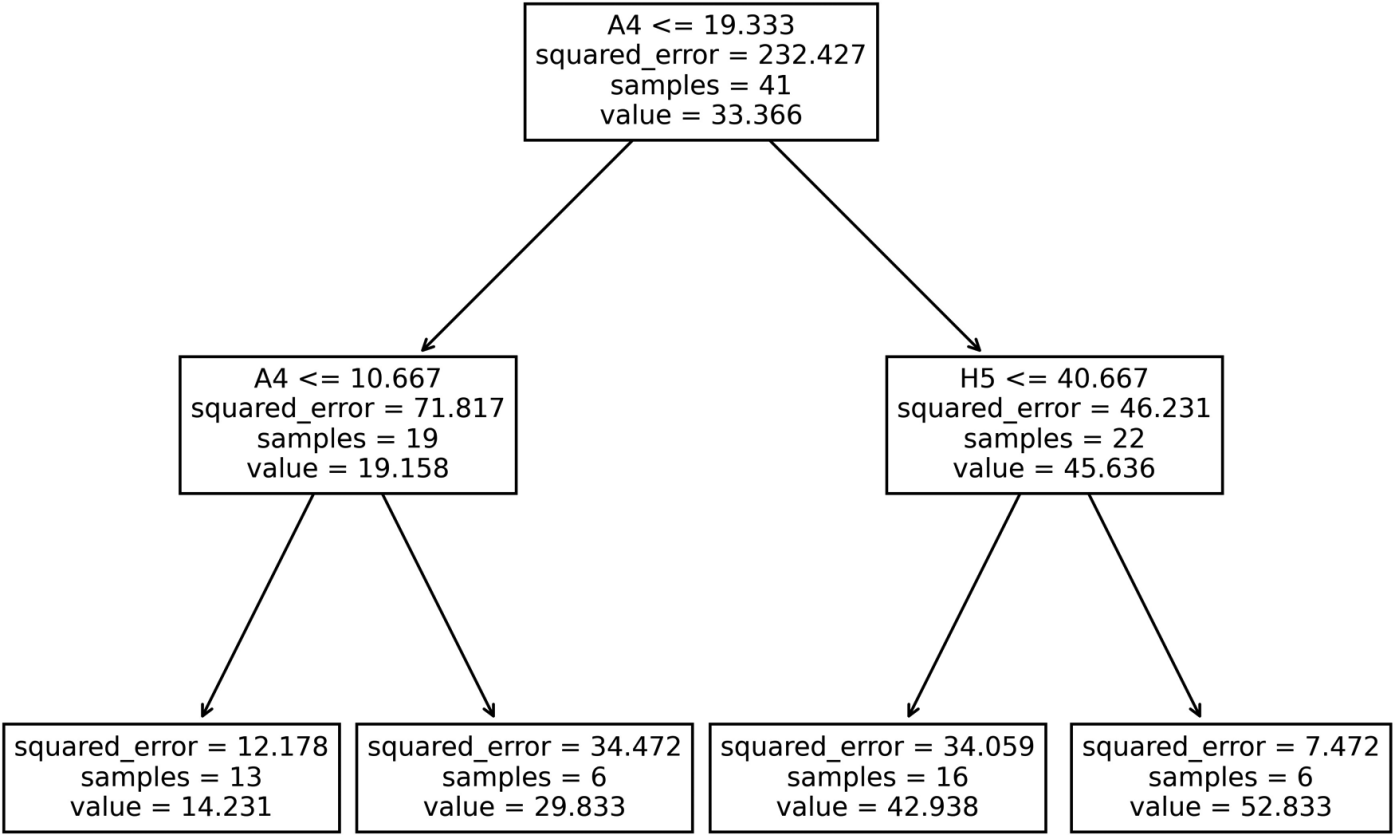
Decision tree generated using participants’ rate of activities performed in the clinic as input features to estimate their fugl-Meyer scores. For each splitting node, if a participant's rate for the specified task (A4: “Reach to Target #2 or H5: “Key Pinch Grip”) was less than or equal to the threshold rate shown, participants were sorted to the left branch, otherwise, they were sorted to the right branch.

From the iterative model generating process (Supplementary Figure S2), the decision tree with maximum allowable depth equal to 2 and minimum samples per leaf equal to 6 was chosen, Figure 6. This model presented improved training fit statistics (r^2^ = 0.89, RMSE = 4.82) over the previous exponential model made with a single activity performed in the clinic (r^2^ = 0.85, RMSE = 6.00). This data-driven process created a model using two of the 12 exercises: “A4: Reach to Target #2” and “H5: Key Pinch Grip”. Patients below a certain performance threshold in the reaching task were sorted to the lower range of the scale, and then again sorted to a high impairment (14 points) or medium impairment (30 points) category by a lower threshold on the same task. Patients exceeding the initial performance threshold for the reaching task, were then evaluated by their ability in a gripping task using their hand, being further sorted to a mild (43 points) or very mild impairment (53 points) category.

## 4. Discussion

In this work, rates of specific exercise activities, captured by a sensorized home stroke rehabilitation system, were used to estimate UEFM scores. The exercises that provided the most utility (“A4: Reach to Target #2” and “H5: Key Pinch Grip”) could theoretically be measured with simple push buttons. Thus, this approach could be replicated with a simple low-cost system that would not require participants to precisely don and doff multiple sensors.

The data here were gathered using a commercially available system that has a demonstrated record of usage across thousands of users (42). This history of usage data suggests that users are able to independently understand and operate the system, which could facilitate automated execution of assessment activities using these models.

The validated models presented here (i.e., the exponential model made with clinic data (r^2^ = 0.83 RMSE = 6.22), the exponential model made with home data (Final Home Model with Final Home Data: r^2^ = 0.69 RMSE = 8.70), and the decision tree made with clinic data (r^2^ = 0.72, RMSE = 7.99)) provide comparable performance to more complicated approaches (Table 1). Additionally, this work presents a model using data from individuals practicing unsupervised in the home whereas most previous uses data taken in a clinical setting.

### 4.1. Insights from the decision tree modeling approach

Out of the 12 exercises performed in the clinic, the exercise with the more predictive power was “A4: Reach to Target #2”. This feature appeared most strongly in the exploratory linear regression heatmap and in the decision tree modeling process. The presented decision tree model uses this reaching task and “H5: Key Pinch Grip” to predict UEFM, which mirrors clinical knowledge that recovery typically starts with proximal ability, such as a gross arm movement, and proceeds to recovery of distal function, such as finer dexterity tasks for the hand (19). As such, the decision tree model provides a data-driven approach that presents explanatory features that match clinical understanding of recovery patterns. Consistent with this idea, prior analyses of the UEFM have confirmed the individual items have a difficulty hierarchy, proceeding from proximal to distal (43, 44). Further, the idea that measurements of a smaller number of movements can be used to predict total UEFM score is consistent with studies that have created shortened versions of the UEFM assessment (45).

### 4.2. Difference between clinic and home performance

The models made with data taken in clinic had higher r^2^ and lower RMSE than the model made with home data likely because there was less variance in the way participants performed their exercises in the clinic than in how they performed exercises at home. In the in-clinic phase of the study, a single therapist standardized the placement of the FitMi sensors, prevented patients from performing exercises with compensation, and set a uniform time limit for each exercise. At home, though the system provided instructions for each exercise, participants were unsupervised, so they may have performed the exercises with compensation and may have changed the placement of the pucks. The system is structured to increase the challenge of the activity as users complete target numbers of repetitions. So in contrast to the clinic scenario where patients perform as many repetitions as they can in a set time limit, the home system sets increasingly difficult target numbers of repetitions over varying durations. This feature may increase user engagement, but it may also add elements of fatigue or may encourage users to employ strategies to pace themselves. Therefore, the rates of participants interacting with the system at home may not be directly comparable to the rates performed in the clinic. Even the presence of a supervising therapist may have been an additional motivator for patients to exert themselves, that would then be absent in the home environment. Some of this variation can be seen in Figure 5A. At home, participants with higher UEFM scores decreased their exercise rate relative to their in-clinic performance, and participants with lower UEFM score increased their exercise rate relative to their in-clinic performance.

### 4.3. Limitations

The sample size used in this study was relatively small (*n* = 41 in-clinic, *n* = 14 at home). Our ability to model data generated at home was further limited by the sparsity and diversity of exercises that participants performed at home. Participants were not given explicit instructions on what exercises to perform at home, which resulted in varying participation across available exercises among participants. Further, in the home setting, without supervision, participants may perform exercises differently than expected. This phenomenon likely contributed to the increased variance. Conversely, for the in-clinic phase of the study, the attending therapist stopped patients if they began performing the exercises with compensation and recorded only the repetitions performed correctly, which meant some participants recorded zero repetitions for some exercises. This led to a y-intercept for the in-clinic model at an UEFM score of 14, such that the model has a floor effect for individuals with UEFM score < 14. During the recruitment, more male patients than female patients were admitted for stroke to the hospital where the trial took place, and through the inclusion screening process, only a few female participants were eligible to participate, able to be contacted, and agreed to participate in the study (*n* = 4). Further, the randomization process was based on UEFM score alone, which resulted in all the female participants being allocated to the control group of the RCT. The participants who used the system at home were all in the subacute phase of stroke recovery. Individuals in the chronic phase of stroke recovery may not fit this model.

### 4.4. Future work

As assessment is an important part of the rehabilitation process, systems designed for home-rehabilitation should aim to incorporate periodic assessments. The present study suggests that incorporating a forward-reaching exercise and measuring rep rate is a simple way to estimate UEFM score. Given sufficient fidelity, such a measurement could potentially be used to inform a healthcare provider of a patient's progress, as justification for institutional reimbursements, or serve as a motivator for individuals pursuing their rehabilitation at home unsupervised. To allow this model to be used as a clinical assessment, further research needs to be conducted to verify the test-retest reliability and the model's sensitivity to changes in exercise rate and UEFM. This will require more data taken in the desired setting (home or clinic) paired with clinical outcome measures.

In the FitMi system studied here, such an assessment could be introduced after individuals complete a set number of activities. To match the scenario created for our clinic data, an assessment should encourage participants to perform as many repetitions as they can in a set period of time and should reinforce the importance of performing the activity correctly without compensation. To limit compensation in an unsupervised home environment, IMUs could be placed on the chest and or arm (46, 47), or a motion capture system could monitor trunk motion (48–50).

Improving models such as the ones presented requires accruing larger sets of ground truth data of clinical measurements and sensorized activity. With enough resources, large trials can be conducted to recruit the participants needed, but commercial rehab system vendors and hospitals may represent an untapped dataset for natural experiments. Commercial vendors could offer video sessions with a therapist to collect clinical data, and hospitals using sensorized systems could work with researchers or developers to pair activity data with clinical measures from electronic health records.

An interesting finding was that the multivariate decision tree modeling approach did not improve model performance. This is likely due to the small sample size. Further, we did not attempt to fit a decision tree model to the home data due to the small sample size and the heterogeneity in the exercises performed. Though the decision tree algorithm could theoretically model a data set where not all participants perform the same exercises, again, a larger sample size would be required to produce reliable results. Other nonlinear modeling techniques could be applied, such as a boosted tree method, but their desirability may be limited because many such models would no longer be explanatory or interpretable.

## 5. Conclusion

In this work, we proposed using the rate of activities completed in a sensorized system to estimate the UEFM. The models presented use a reaching task and a gripping task, and the models captured approximately 70% of the variance in UEFM in our data. This approach could be replicated with simple push button systems that do not require participants to precisely don and doff multiple sensors and could be performed unsupervised in the home setting.

## Data Availability

The data analyzed in this study is subject to the following licenses/restrictions: The data analyzed in this study was obtained from Flint Rehab, LLC. Requests to access these datasets should be directed to Daniel K. Zondervan, dzondervan@flintrehab.com.
