# Supplementary material for: Exercise repetition rate measured with simple sensors at home can be used to estimate Upper Extremity Fugl-Meyer score after stroke
Source: Front Rehabil Sci. 2023 Jun 19;4:1181766. doi: 10.3389/fresc.2023.1181766 (PMC10315847; doi:10.3389/fresc.2023.1181766)
Supplement: Supplementary file 1 [file Datasheet1.pdf]

REHABSTUDIO

Exercise Library

Hand Exercises

H1: Wrist Flexion

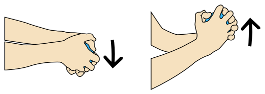

H2: Wrist Deviation

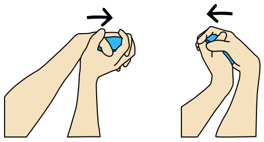

H3: Gripping

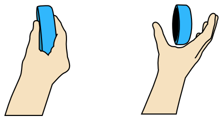

H4: Rotation

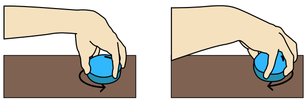

H5: Key Pinch Grip

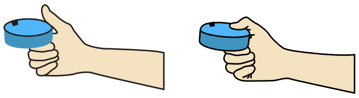

Arm Exercises

A1: Touches

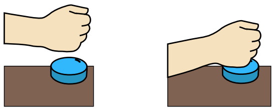

A2: Reach to Target #1

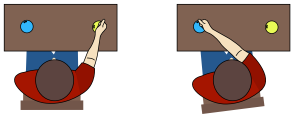

A3: Clapping

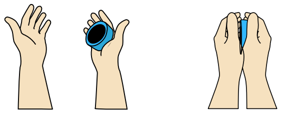

A4: Reach to Target #2

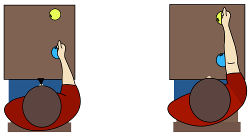

A5: Reach to Target #3

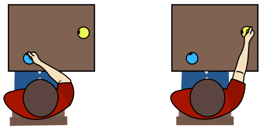

Core Exercises

C1: Weight Bearing - Near

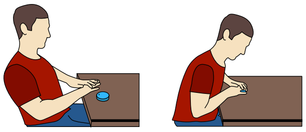

C2: Weight Bearing - Left

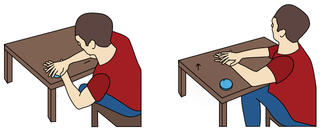

C3: Weight Bearing - Right

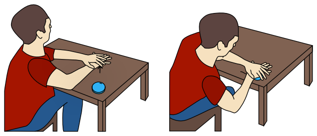

C4: Twists

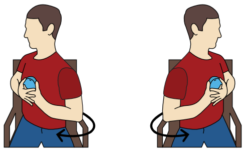

C5: Table Edge Lean

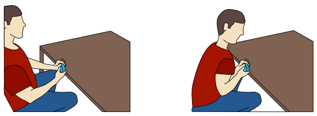

Leg Exercises

L1: Stomps

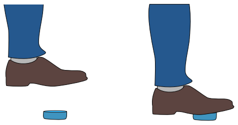

L2: Knee Squeeze

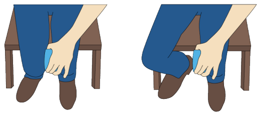

L3: Step to Target #1

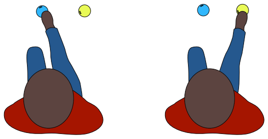

L4: Toe Taps

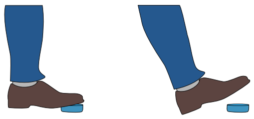

L5: Marching

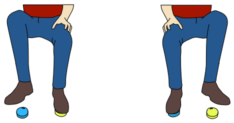

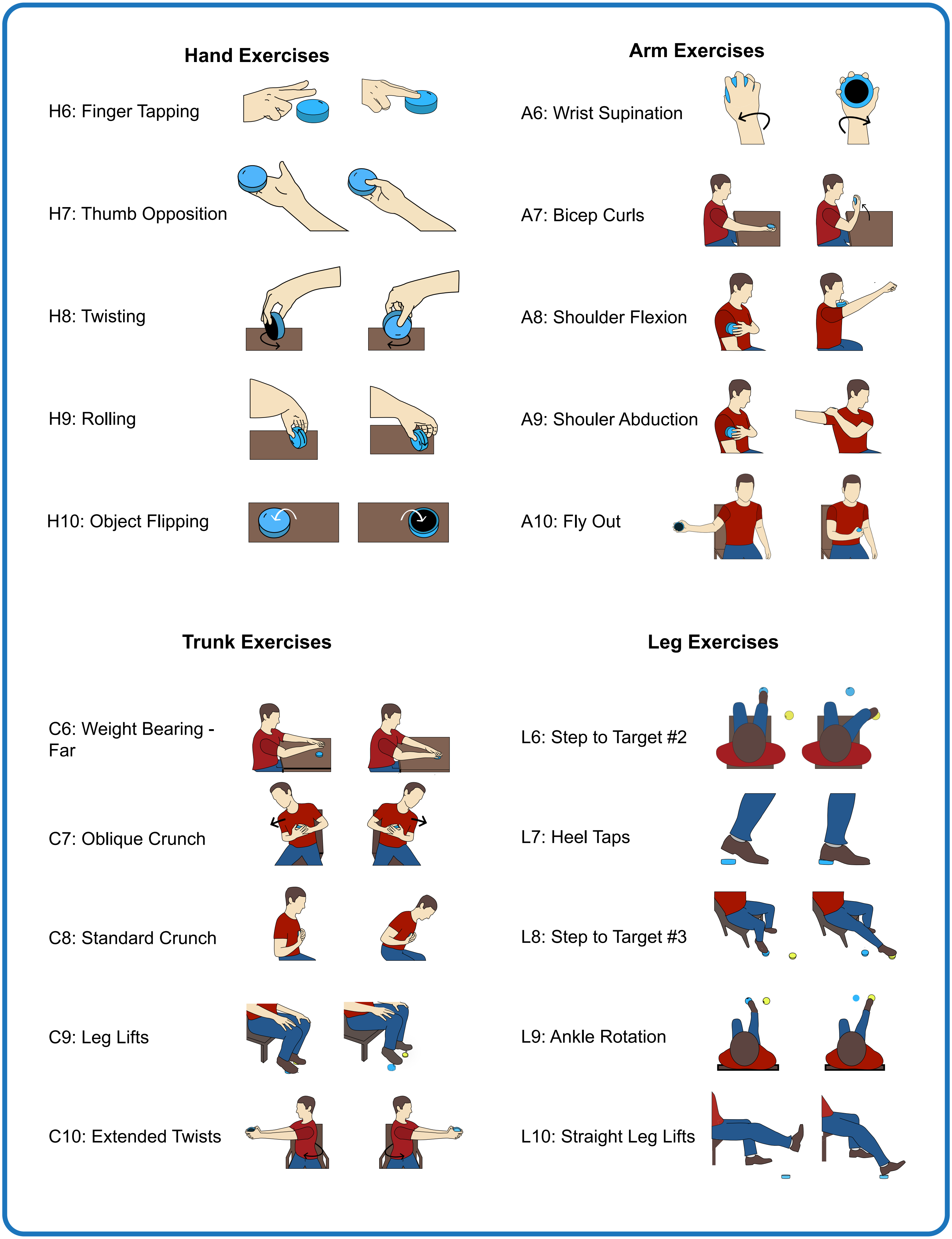
Hand Exercises
H6: Finger Tapping
H7: Thumb Opposition
H8: Twisting
H9: Rolling
H10: Object Flipping
Arm Exercises
A6: Wrist Supination
A7: Bicep Curls
A8: Shoulder Flexion
A9: Shouler Abduction
A10: Fly Out
Trunk Exercises
C6: Weight Bearing - Far
C7: Oblique Crunch
C8: Standard Crunch
C9: Leg Lifts
C10: Extended Twists
Leg Exercises
L6: Step to Target #2
L7: Heel Taps
L8: Step to Target #3
L9: Ankle Rotation
L10: Straight Leg Lifts
