# Supplementary material for: Exercise repetition rate measured with simple sensors at home can be used to estimate Upper Extremity Fugl-Meyer score after stroke
Source: Front Rehabil Sci. 2023 Jun 19;4:1181766. doi: 10.3389/fresc.2023.1181766 (PMC10315847; doi:10.3389/fresc.2023.1181766)
Supplement: Supplementary file 2 [file Datasheet2.docx]

Supplemental Figure 1

Exercise Repetition Rate Measured with Simple Sensors at Home can be used to Estimate Upper Extremity Fugl-Meyer Score after Stroke

Veronica A. Swanson^*^, Christopher Johnson, Daniel K. Zondervan, Susan Shaw, David J. Reinkensmeyer

*** Correspondence:** Veronica A. Swanson: swansonv@uci.edu


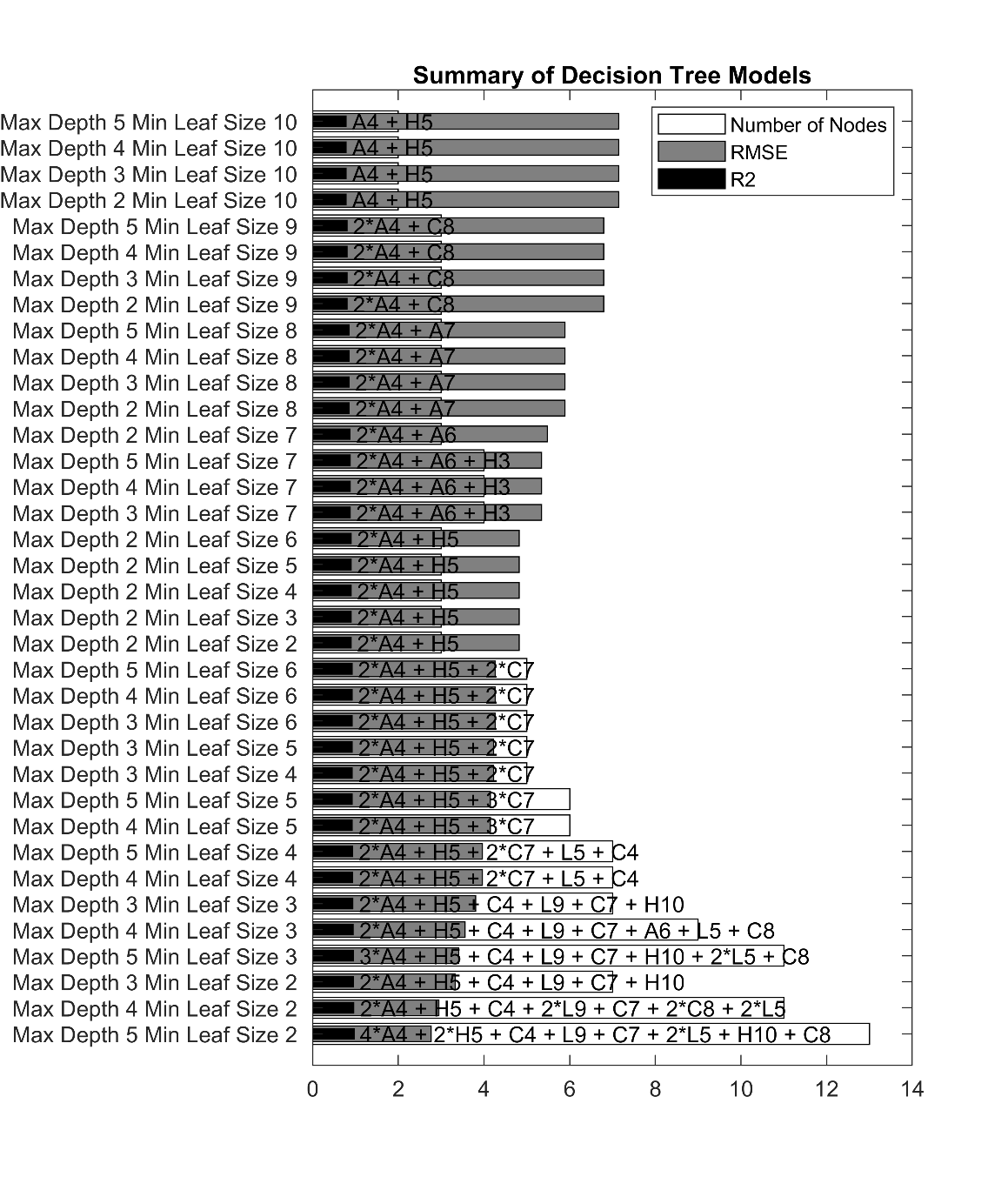


**Supplementary Figure 1.** To prevent overfitting the decision tree, multiple models were fit with varying maximum allowable depths and minimum samples per leaf (i.e. prediction node), where increasing the depth and reducing the minimum samples per leaf results in models with increasing accuracy but also increasing potential for overfitting. Models were compared by the research team considering the complexity of the resulting model, the depth and leaf design criteria, the RMSE, and the r^2^. For each model, this Figure displays the number of nodes, the RMSE, and the r^2^. In the text is printed the exercises used within the nodes of each model, preceded by a scalar coefficient if that exercise was used more than once. This visualization allowed the researchers to graphically optimized the tree, ultimately selecting a tree with Max Depth 2 and Minimum Leaf Size 6.
